# Twitter mobility dynamics during the COVID-19 pandemic: A case study of London

**DOI:** 10.1371/journal.pone.0284902

**Published:** 2023-04-26

**Authors:** Chen Zhong, Robin Morphet, Mitsuo Yoshida

**Affiliations:** 1 Center for Advanced Spatial Analysis, University College London, London, United Kingdom; 2 Institute of Business Sciences, University of Tsukuba, Tsukuba, Japan; University of Calabria: Universita della Calabria, ITALY

## Abstract

The current COVID-19 pandemic has profoundly impacted people’s lifestyles and travel behaviours, which may persist post-pandemic. An effective monitoring tool that allows us to track the level of change is vital for controlling viral transmission, predicting travel and activity demand and, in the long term, for economic recovery. In this paper, we propose a set of Twitter mobility indices to explore and visualise changes in people’s travel and activity patterns, demonstrated through a case study of London. We collected over 2.3 million geotagged tweets in the Great London Area (GLA) from Jan 2019 –Feb 2021. From these, we extracted daily trips, origin-destination matrices, and spatial networks. Mobility indices were computed based on these, with the year 2019 as a pre-Covid baseline. We found that in London, (1) People are making fewer but longer trips since March 2020. (2) In 2020, travellers showed comparatively reduced interest in central and sub-central activity locations compared to those in outer areas, whereas, in 2021, there is a sign of a return to the old norm. (3) Contrary to some relevant literature on mobility and virus transmission, we found a poor spatial relationship at the Middle Layer Super Output Area (MSOA) level between reported COVID-19 cases and Twitter mobility. It indicated that daily trips detected from geotweets and their most likely associated social, exercise and commercial activities are not critical causes for disease transmission in London. Aware of the data limitations, we also discuss the representativeness of Twitter mobility by comparing our proposed measures to more established mobility indices. Overall, we conclude that mobility patterns obtained from geo-tweets are valuable for continuously monitoring urban changes at a fine spatiotemporal scale.

## 1 Introduction

The World Health Organization (WHO) officially declared the SARS-CoV-2 outbreak a Public Health Emergency of International Concern on the 30th of January 2020 and a Global Pandemic on the 11th of March 2020. WHO urged countries to adopt strict social distancing and quarantine measures to prevent virus spread and protect the public health [1]. Despite international efforts to contain the transmission, coronavirus has spread worldwide, resulting in more than 130 million cases and 2.8 million deaths as of March 2021, with more than 700,000 cases tested positive, and 18,000 Coronavirus (Covid-19) related deaths occurred in London alone [2], and this is likely to continue increasing, although at a reduced rate. To mitigate these effects, measures like social distancing and lockdown have been imposed worldwide. In the UK, the initial lockdown began on the 23rd of March 2020. To promote economic recovery, the government encouraged people to go back to work on the 9th of July 2020. Subsequent lockdowns were enforced from the 5th of November to the 2nd of December, given the second wave and again from the 5th of January 2021. Each lockdown has prompted a significant change in human behaviour. Substantial evidence has shown profound changes in people’s lifestyles and travel behaviours, which may persist post-pandemic [3]. How the movement of people has accelerated the virus transmission, and in reverse, how much Covid measures (i.e., travel quarantine, border control) have impacted people’s movement has been broadly investigated by incorporating human movement data into various analytical models [4–7], with a good number of studies conducted to project the impact of travel limitations at national and international scales [5, 6, 8–10]. For instance, Chinazzi, Davis [6] developed a global metapopulation disease transmission model and found the travel quarantine of Wuhan delayed the overall epidemic progression significantly on the international scale compared to that at the level of the Wuhan local area. Most of the relevant studies at a global scale used aviation networks, which are relatively coarse datasets. The nationwide movements were tracked using finer human movement data. Thus travel between Wuhan and the other cities in China was tracked using mobile phone data and revealed a strong correlation between total population flow and the number of infections at the city level [10]. The impact of COVID-19 on mobility also varies over time. The disease spreading process consisted of a significant inter-city diffusion before the Chinese New Year and a subsequent intra-city diffusion after the Chinese New Year [7].

In such a context, cost-effective and easily accessible approaches for monitoring continuous changes in disease transmission and human mobility become essential for addressing people’s social needs and travel demands in the short term and for securing economic recovery in the longer term. Other than frequently updated surveys, efforts have been made to develop alternative, less time-consuming data sources and approaches. The mobile app—NHS COVID-19 is designed for contact tracing and sending alerts to people who have visited a venue where they may have come into contact with Coronavirus [11]. The ZOE COVID Symptom Study app collects daily health reports from volunteers. It is potentially a good example of Volunteered Geographic Information (VGI) [12]. With big crowdsourced data, ZOE is claimed to provide insights into how the pandemic is changing up to five days faster than the official government figures [13]. Companies such as Google, Apple and Citymapper have released their aggregated and anonymised community mobility reports based on location data generated from their mapping services. Public authorities such as Transport for London (TfL) are also updating their reports on public transport usage regularly on a more frequent basis. These Coronavirus mobility report was generated based on these reports and compared to a pre-pandemic baseline [14]. As the COVID-19 pandemic continues worldwide, an unprecedented amount of mobility data, including social media data and aggregated mobile phone data, has been accumulated and released to empower research communities’ collaborative research [5, 15–18].

Compared to the other types of human location data, Twitter data is open to the public (particularly if using a streaming Application Programming Interface (API)), is less privacy sensitive and is highly available in most cities. Furthermore, these established mobility reports, e.g., the Google mobility report, only provide aggregated and relative figures. Twitter data comes in its original data format with latitude and longitude. This gives the flexibility of analysis and cross-validation at various spatial and temporal scales. Various biases remain; however, for instance, the number of geotagged tweets is low compared to the total number of tweets and varies among countries [19] but the more extended temporal frames could compensate for the spatial sparsity of sample points for analysing human mobility and activity patterns [19, 20]. Geolocated Twitter data has proved helpful in proxying mobility patterns globally [21] and regionally [22]. The sizeable daily number of tweets accumulating over time has already been experimented with for mapping urban areas, inferring activity types, and proxying urban flows in various urban contexts worldwide. -Lenormand, Tugores [23] analysed tweets in 39 countries and showed a positive correlation between the number of tweets on the road and the Average Annual Daily Traffic on highways in France and the UK, showing the possibility of using tweets for mapping mobility and infrastructure. Verification has been conducted on origin and destination data extracted from tweets. It was concluded that it is possible to produce reliable estimates of temporal mobility flows on weekdays compared with the community surveys [24]. Steiger, Westerholt [25] explored the semantic association between georeferenced tweets and found that the clustering of tweets has a strong positive correlation with workplace population census data. In [26], an analytical framework was proposed to identify spatial clusters at different levels of aggregation and found geotweets a valuable data source for profiling urban structures. Finally, geo-tweets are largely available, even in the Global South, and have been used as an alternative data source for estimating flows of people among counties in Kenya [27] and evaluating the built-environment for urban planning purposes [28].

Continuous research progress on Twitter data analysis has provided valuable insights into the current issue—COVID-19. One stream of Twitter data analysis focuses on the emotional and mental responses to COVID-19 utilising the textual information in tweets [29, 30]. Another stream makes use of location information to detect changes in mobility patterns impacted by social distancing [31, 32] and to track the spread of disease [8]. Twitter-based mobility data has been proposed in various forms to monitor human mobility dynamics during the COVID-19 pandemic [9, 33], with case studies predominantly in the US [32, 34] or globally at a regional scale [8]. This paper contributes an updated view on the case study of London, with the aim of answering the following questions:

**(Q1) is there any strong relationship between the identified changes in Twitter mobility and changes in the incidence and prevalence of** COVID-19**?**

Going beyond one case study, our research seeks answers to a broader question regarding the potential and limitation of Twitter data. Aware of the sparsity and bias of Twitter data, we ask:

**(Q2) can we still use geotweets to monitor urban changes at smaller than city-scale?**

**(Q3) can location-based mobility index computed from geo-tweets be an effective approach to capture the collective effects of mobility changes?** (e.g., increased/decreased local attractiveness caused by changes of activity location choices)

To answer the above three questions, we extracted daily trips from geotagged tweets and compared them to pre-Covid baselines. Global changes were profiled by Twitter mobility indices, i.e., the intensity of travel and average travel distance. Twitter mobility in this study does not refer to journey to work mobility but rather to more localised interaction. Therefore, based on this, local changes were identified by local attractiveness and community structure at the level of Middle Layer Super Output Area (MSOA). While MSOA are zones designed to contain 5,000 to 15,000 residents and 2,000 to 6,000 households [35] and are much smaller spatial units than regions and nations used in most of the earlier studies. These Twitter mobility indices were related to COVID-19 cases to investigate if any aspatial or spatial relationship exists over time. To further understand the representativeness of geo-tweets, we compared them to the mobility index used in the official London mobility report [14]. It is worth noting that all measures used in this paper are implemented by established Python packages and shared on GitHub, with the aim of developing a highly reproducible methodology that can be easily applied in other cities for international comparison and collaborative research.

## 2 Materials

The geo-tweets used for this study were acquired through the Twitter streaming API between Jan 2019 and Feb 2021. Any tweets with the attribute of country coded as the UK were collected, regardless of user profiles and the hashtag of the tweets and filtered further to include only locations within the Greater London Area (GLA). In particular, to crop the geotweets within the GLA, we used latitude and longitude rather than other locational tags, e.g., place name, since latitude and longitude gives greater accuracy. Therefore, by geotweets, we refer to tweets with valid values of latitude and longitude. Our data sets cover a long enough period of time to uncover a non-monotonic trend, which is generally in line with the UK COVID-19 timeline.

The initial lockdown in the UK began on the 23rd of March 2020. Measures were relaxed over time since July 2020 as the government re-evaluated the COVID-19 situation and encouraged activities for economic recovery. The second wave of COVID-19 hit the UK in November. Subsequent lockdowns prevailed from the 5th of November to the 2nd of December and then started again from the 5th of January 2021. In total, initially, there were about 1.3 million geo-tweets collected for 2019, 1 million geo-tweets collected for 2020, and 0.12 million geo-tweets collected in the first two months of 2021. Similar data cleaning rules were applied to those used in our previous paper concerning Twitter in London [26]. Thus, Twitter accounts posting an abnormal number of tweets greater than average by a standard deviation are likely to be fake users or commercial accounts and have therefore been removed. Furthermore, a trip requires displacement between at least two distinguishable location points with the same user id. Therefore, any tweets posted at the same coordinates are considered static users were filtered out in the follow-up process of extracting daily trips. The historical COVID-19 related data was obtained from the official UK government website (https://coronavirus.data.gov.uk). The official coronavirus dashboard reports the progress of the coronavirus pandemic as an up to date and authoritative summary of key information. Weekly rolling sums and population-based rates of new cases by specimen date time-series data are open to the public and available to download at the MSOA level.

**Fig 1:**
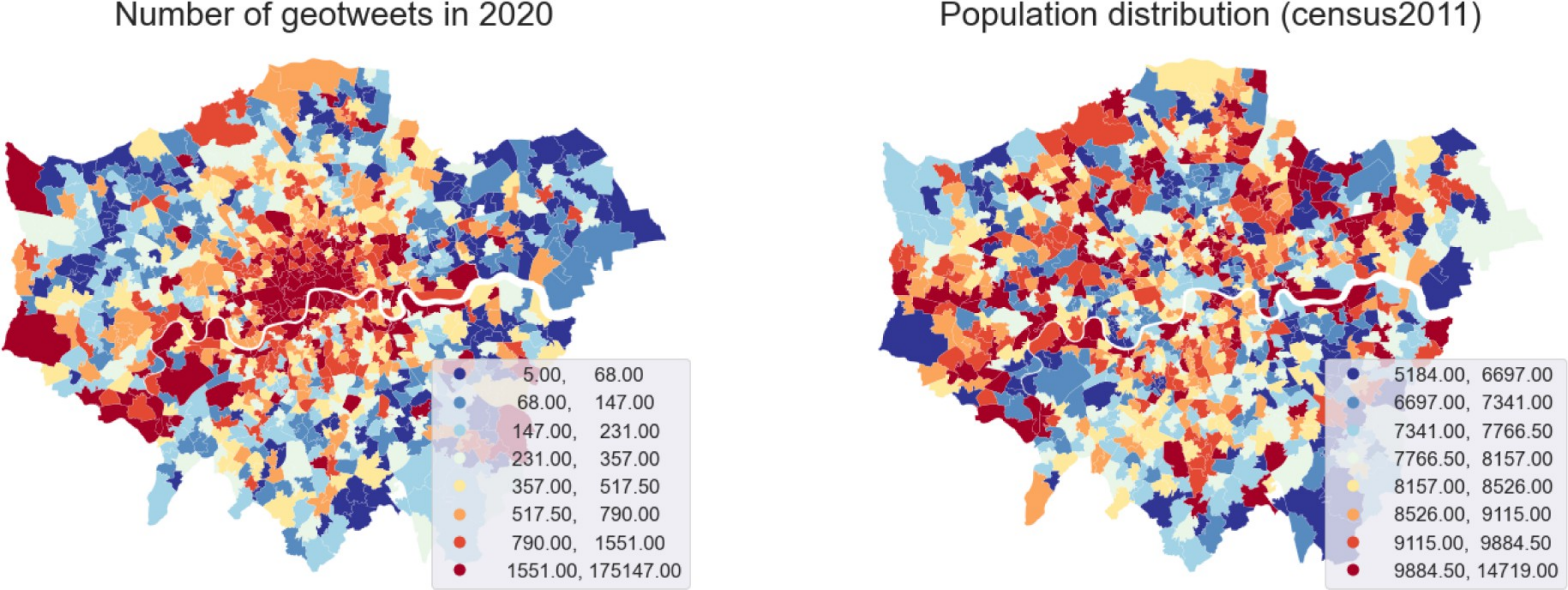
Number of geotweets collected in 2020 (left) and the number of residents reported in UK census 2011 (right). Further statistics on the number of geotweets and intensity of twitter mobility at the MSOA level can be found in figures in **S1** and **S2** Figs.

**Fig 2:**
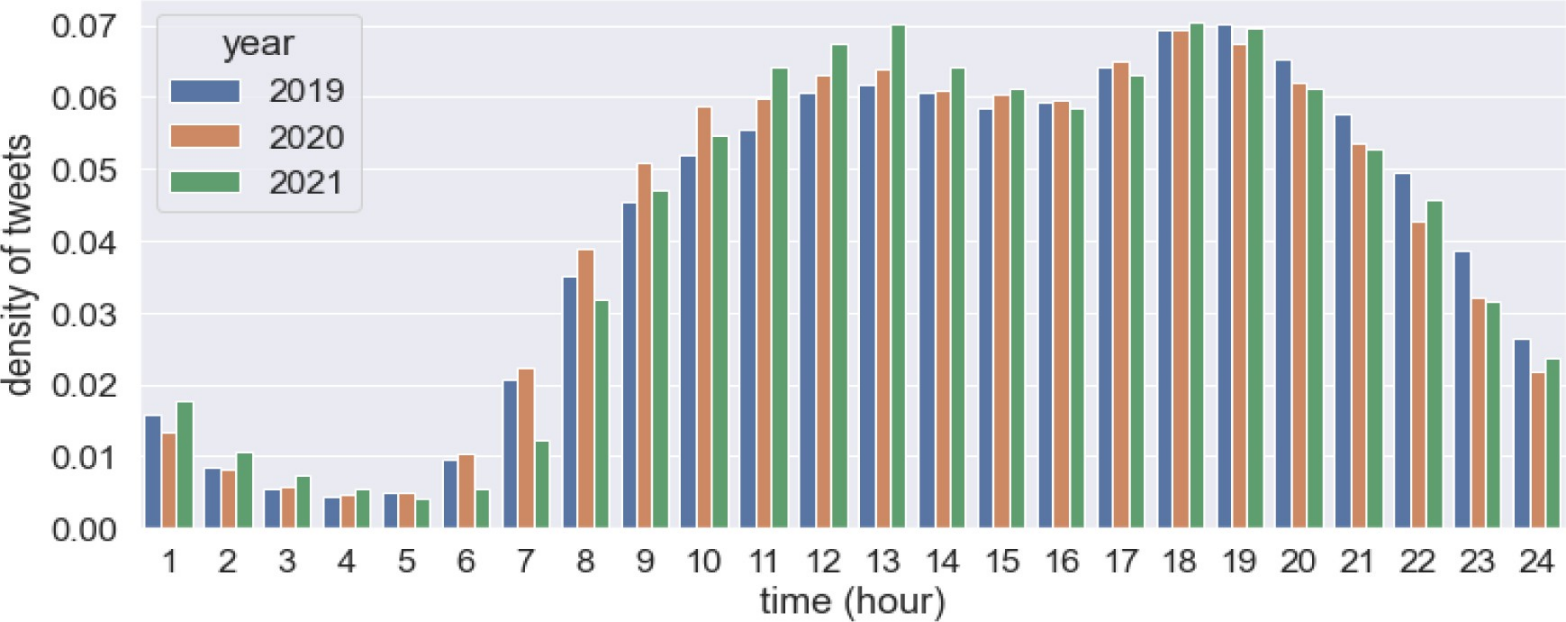
Temporal distribution of geo-tweets in 2019, 2020 and 2021.

Table 1 reports basic figures for the processed geo-tweets. The statistics show the average number of tweets, users and extracted trips on an average day, including weekends, weekdays and public holidays. We can already discern some changes from these basic figures. The total number of tweets is reducing, and fewer travellers are detected. To give a better sense of the Twitter geography, we have mapped the spatial distribution of tweets in 2020 and contrasted it to the residential population distribution in Fig 1. There is barely any correlation between the two, indicating geotweets are located in activity places rather than in home locations. The overall spatial distribution remains the same over the years with a big cluster in the west end city centre and scattered sub-clusters in town centres. The temporal distribution of tweets in the three years (shown in Fig 2) show very similar shapes with clear morning and afternoon peaks, despite there being a slightly larger morning peak in 2020 and 2021, which is possibly caused by the new mode of working from home for which people have adjusted routing schedules accordingly (e.g. activities start earlier because of less time on the road; no need for staggered shifts to avoid traffic jams).

**Table 1:** Basic statistics of geo-tweets in London on an average day.

*Year*	*Number of users*	*Avg*. *tweets per user*	*Number of travelled users*	*Avg*. *tweets per travelled user*	*Average number of travelled locations*	*Average travelled distance (meters)*	*Ratio of travelled Twitter user*
**2019**	1653	1.28	120.58	3.05	2.32	5426.56	0.073
**2020**	1212	1.27	64.96	2.87	2.22	6405.10	0.051
**2021**	854	1.29	40.25	2.96	2.22	6797.59	0.047

## 3 Methods

**Fig 3:**
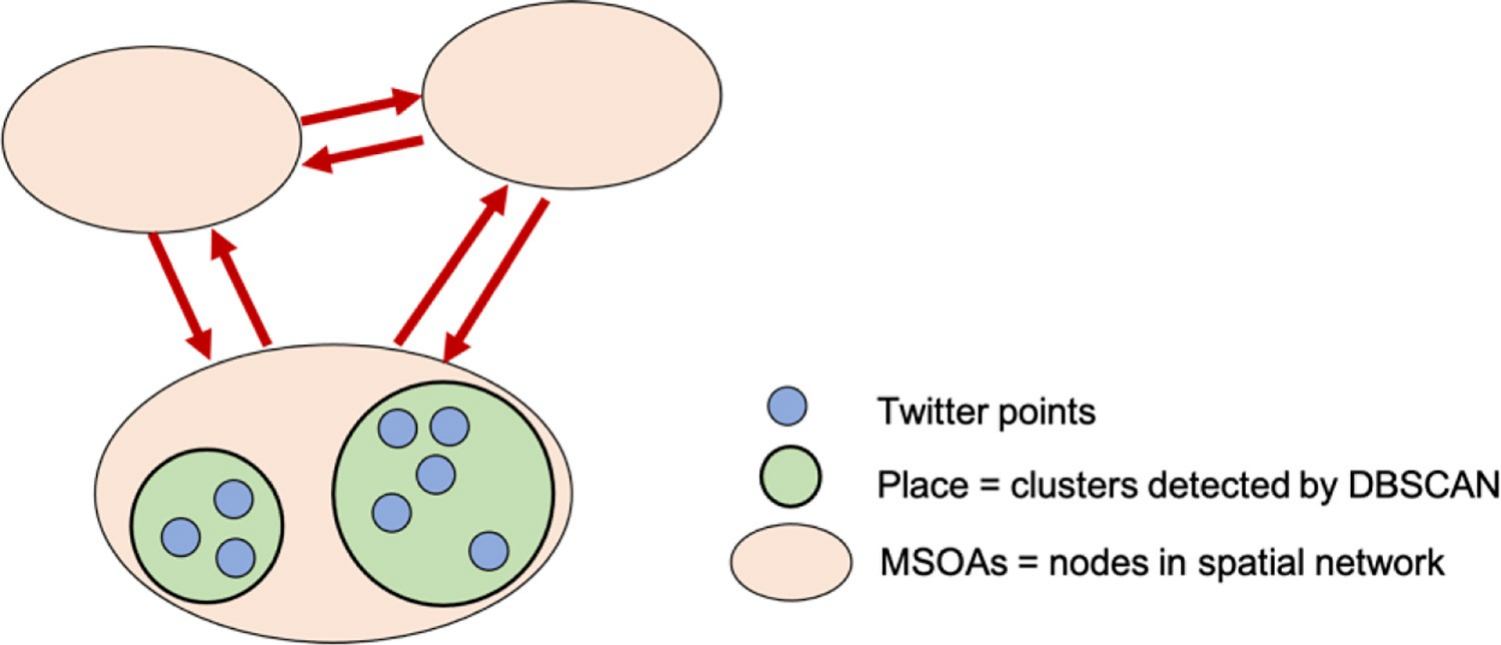
From geo-tweets to a spatial network of urban mobility.

This section presents the analytical workflow and detailed techniques used in this study. It is noted that data were collected in accordance with the Twitter Terms of Service and reviewed by the Data Protection Team. The original Twitter points were grouped into a cluster as that illustrated in Fig 3, which is considered a place within an MSOA. Middle Layer Super Output Areas (MSOA) are a geographic hierarchy designed to improve the reporting of small area statistics in England and Wales [36]. Further information about the clustering is detailed in section 3.1. Displacement among detected places within a 24-hour time window is considered a daily trip. These trips were summarised into weekly origin-destination matrices between MSOA zones, which were then converted to a spatial network of flows. Centrality measures were applied to the networks as a proxy of local attractiveness, as detailed in section 3.2. Finally, to investigate the relationship between Twitter mobility and COVID-19 cases, aspatial and spatial correlation analysis was applied and is presented in section 3.3. All experiments, including the performance analysis, were implemented in Python using established packages (i.e., Scipy, Sklearn, Pysal and NetworkX).

### 3.1. Extracting daily trips from geo-tweets

Any displacement between two places that happened within a 24 hour time window was considered a daily trip. Original Twitter points are not equivalent to places because Twitter points with slightly shuffled coordinates are highly likely to be associated with the same place (i.e., people tweeting in the same building or in a park). A place, in our case, is defined as the centroid point of an area where Twitter points are densely distributed. It can be easily identified by a cluster analysis that groups geographically close points together. Various clustering methods are available for the task, but the algorithm most closely aligned with the requirements stated above is DBSCAN (Density-based spatial clustering of applications with noise) [37]. The DBSCAN method requires the specification of two parameters related to the required density of data points that constitute a cluster. The first is a definition of the minimum number of points (minPt) to form a cluster set to 1 in our case, considering the sparsity of points. The second is a specification of the geographical distance (dist) threshold within which two points would be classified as the same place. The threshold is set to be 500 meters, which has often been considered as a walkable distance in urban design and transport-related studies [38, 39]. We have tested different settings which generated slightly varied results. However, that does not significantly impact the final analysis which uses relative rather than absolute, values. Therefore, optimisation of these two pre-specified parameters is not included in this work. As long as the setting is consistent for data in different years, the comparison should be fair and valid. The detected places may be represented by slightly shuffled centroids. That should not give rise to any further uncertainties, because all centroids will be aggregated to bigger spatial units, i.e., MSOA zones, for further analysis. We define two basic indices of daily trips to describe global changes over time. They are the ratio of moving Twitter users to total Twitter users, denoted as *m*_*rate*, and the average travel distance denoted as *avg*_*dis*. The former tells how actively people are making journeys, and the latter is a simple proxy of the threshold of activity space.


$$m\_rate=\frac{m\_ids}{n\_ids}$$ (1)

$$avg\_dis=\frac{\sum_{i=0}^{m}\frac{dis\_trip_{i}}{n\_trips\_m_{i}}}{m\_ids}$$ (2)

Where *m*_*ids* is the number of unique Twitter ids, *n*_*ids* is the number of ids tweeted from at least two different places identified by DBSCAN and therefore, considered as having made at least 1 trip in a day. *n*_*trips*_*m*_*i*_ is the number of trips made by user *i*, *dis*_*trip*_*i*_ is the crow-fly distance of each trip.

### 3.2. Measuring local attractiveness by spatial network analysis

Urban areas and flows can be considered as nodes and edges that together construct a network representing spatial interactions. In recent years, spatial network analysis has been widely applied in mobility analysis to uncover the hidden information of movements and spatial interactions [40–44]. The same type of analysis is employed in this work to quantify local attractiveness and connectivity. Considering that the openly accessed COVID-19 data at the MSOA level is only available weekly, and the distribution of daily Geo-tweets is too sparse in some areas, trips were aggregated by weeks to form weekly flow matrices. From these, directed networks are generated. The nodes of the networks are MSOA zones; edges are flows between the zones; the weight of the edges is the number of trips from one zone to another. Various centrality measures (i.e., betweenness centrality, eigenvector, degree) were applied to the spatial network to understand the functioning of individual zones. At the global scale, community detection reveals the hidden relations among the nodes in the network. All the analyses use established algorithms that are available in commonly used Python/R packages (e.g., NextworkX, igraph). The mathematics underlying these analysis can be found in the related works of complex network analysis [45].

Local attractiveness measures how much an area has been visited. It can be proxied by various centrality measures which emphasise different aspects of centrality. For instance, Eigenvector centrality is often used to identify urban hubs as areas that can get information to many other areas quickly. Betweenness centrality is used to identify critical areas that connect two otherwise disparate parts of a network [42, 44]. Since the results of these centrality measures display a similar trend, we present degree centrality as the most straightforward measure of local attractiveness because it can be easily interpreted as zones that attract comparatively more visits and is computed as

$$A(i,t)=\frac{\sum D(i,j,t)}{\sum D(j,t)}$$ (3)

Where attractiveness at zone *i* during period *t*, is the number of connections to each area *j*.

Besides a weekly profile of individual zones, community detection is applied to investigate how closely urban areas are connected to the other parts of the city. The most commonly used Modularity-based communities algorithm is applied, which operates by optimising modularity to ensure intra-trips of each community outnumber inter-trips between communities [46].

### 3.3. Spatial and temporal correlation analysis of Twitter mobility and COVID-19 cases

Based on the Twitter mobility indices computed in section 3.1. and 3.2., we investigate the relationship between COVID-19 cases and urban mobility at the local level over the study period. Correlation analysis, i.e., Moran’s I [47] and Bivariate Moran’s I [48], were applied to investigate the relationship. The former checks whether any spatial autocorrelation exists in the distribution of reported COVID-19 cases and local attractiveness, respectively. Bivariate Moran’s I is a global measure of spatial autocorrelation to estimate the influence one variable has on the occurrence of another variable in close proximity [48]. It is applied to investigate the relationship between the local attractiveness and the reported cases of COVID-19 19. Since both data contains spatial and temporal dimensions, the problem could naturally be classified as a space-time regression. However, in our case, neither COVID-19 cases nor the Twitter mobility index (shown in Fig 5) exhibit a simple monotonic trend. Instead, variability caused by COVID-19 measures (i.e., lockdown, social distancing) is clearly observed. The variability indicates the relationship between the two is also changing over time. With this consideration, we aggregated the trips into three periods, namely, March 2020 –June 2020, July 2020 –October 2020, November 2020 –Feb 2021, as aforementioned, roughly in line with the timeline of UK coronavirus lockdowns.

## 4 Results

### 4.1. Changes in travel behaviour during COVID-19

**Fig 4:**
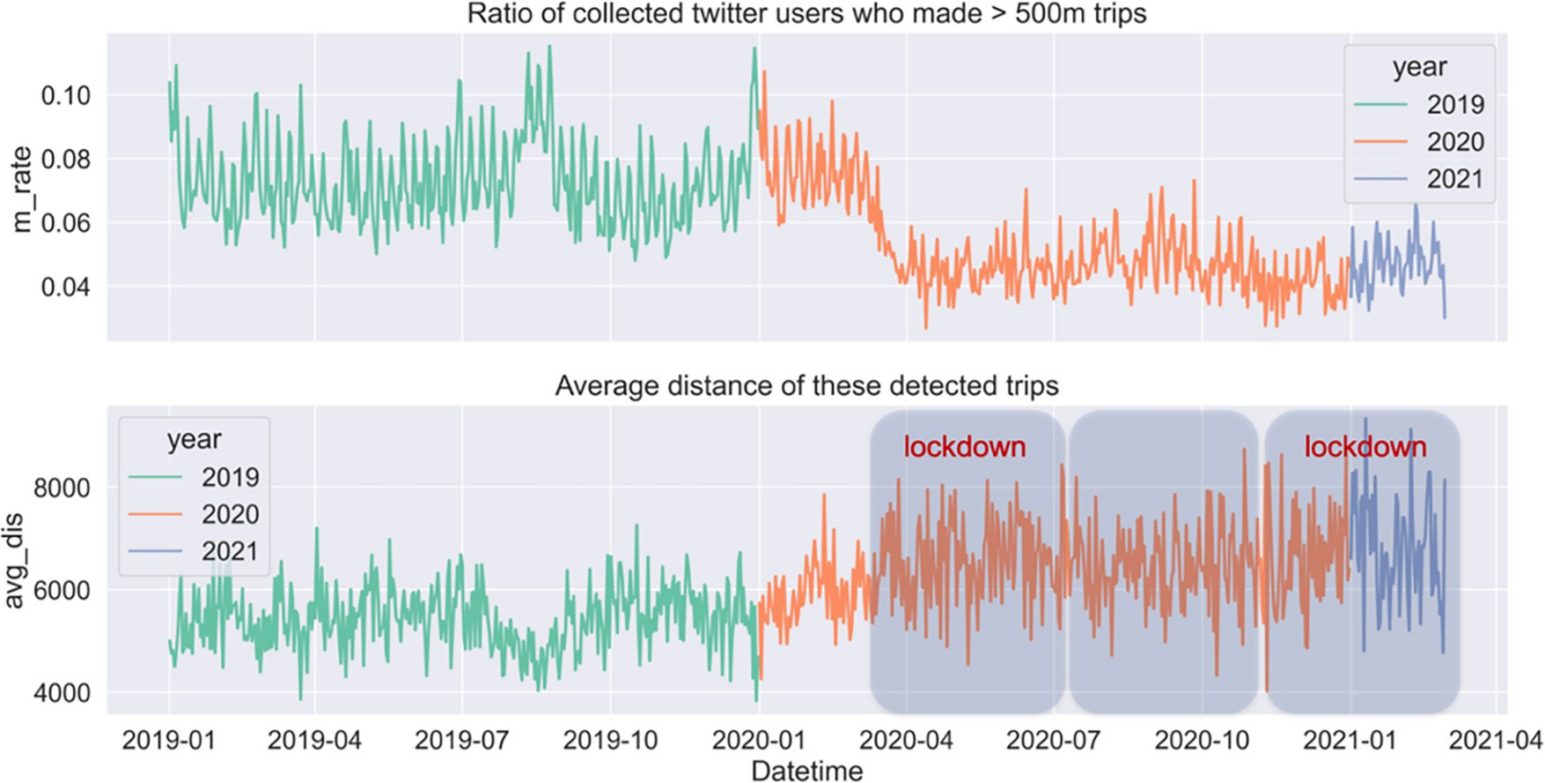
Twitter mobility indices (i.e., m_rate, avg_dis) over the study period.

**Fig 5:**
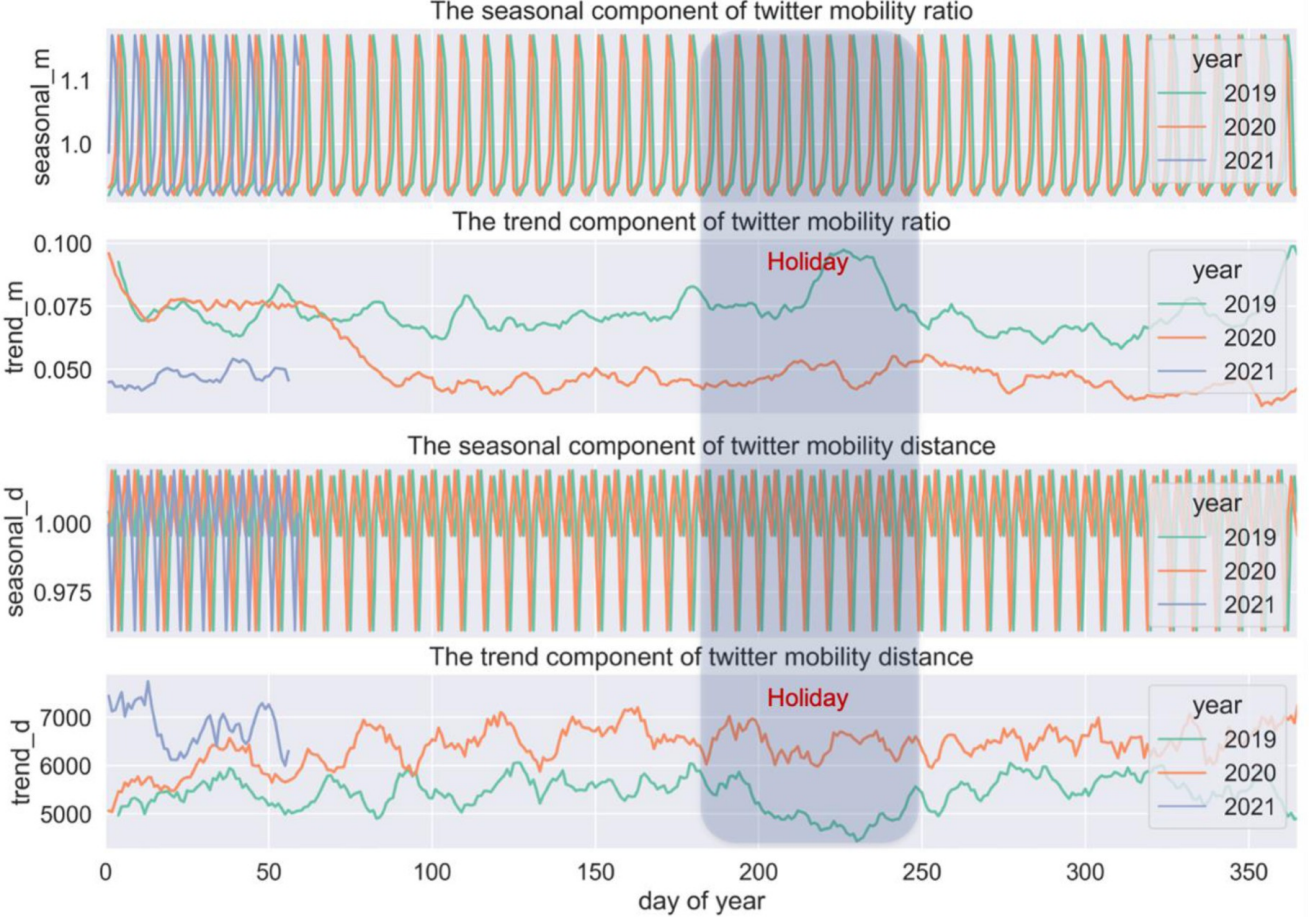
Trends and seasonality in Twitter mobility patterns.

Fig 4 shows clear changes in both aspects (i.e., *m_rate* and *avg_dis*) that are well in line with the timeline of lockdown. Decomposition is applied to temporal profiles in order to generate a clearer view of trends and seasonality in mobility patterns. The component of the trend shows the overall changes in travel intensity and distance. The component of seasonality could be seen as a validation of the detected patterns, which are expected to be stable and unchanged over time. The decomposed results are shown in Fig 5. Compared to the same period in the year 2019. The ratio of travellers decreased dramatically immediately after March 2020 and yet was back to normal levels by the time this research was conducted (Fig 4, top); and people began to travel for a longer distance than pre-Covid (Fig 4, bottom). people began to travel for a longer distance than pre-Covid (Fig 4, bottom). It could be explained by people having had to make substantial adjustments to their daily routine and travel chains due to Covid measures, as evidenced in [49]. In particular, many people choose to work from home [50]. Some have relocated to outer area. Overall, the housing market exhibit a dispersed choice [51]. People were commuting on fewer days per week and travelling further to work. Subsequently, non-work-related trips, which used to happen near working or residential locations and chained to commuting trips, were rearranged and made to someplace else, resulting in more extended travel distances. More discussions on travel purposes and activity location are discussed in the following section. The seasonal component of the temporal profile and the hourly profile in Fig 2 show consistent daily and weekly cycles over three years. This means that although people may tweet at different locations and make fewer trips, the temporal dimension of tweeting and mobility behaviour does not exhibit any significant changes. There is quite the opposite trend between the year 2019 and 2020 between June and August, which is usually the holiday season. It is when overseas visitors temporarily stay in London and contribute a good portion of the daily social and entertainment trips, whilst London residents travel somewhere else. However, these kinds of flow exchange did not happen in 2020, mainly due to the concern with virus transmissions, travel restriction and border control. Consequently, the peak shown in 2019 disappeared in 2020.

### 4.2. Changes in location choice during COVID-19

**Fig 6:**
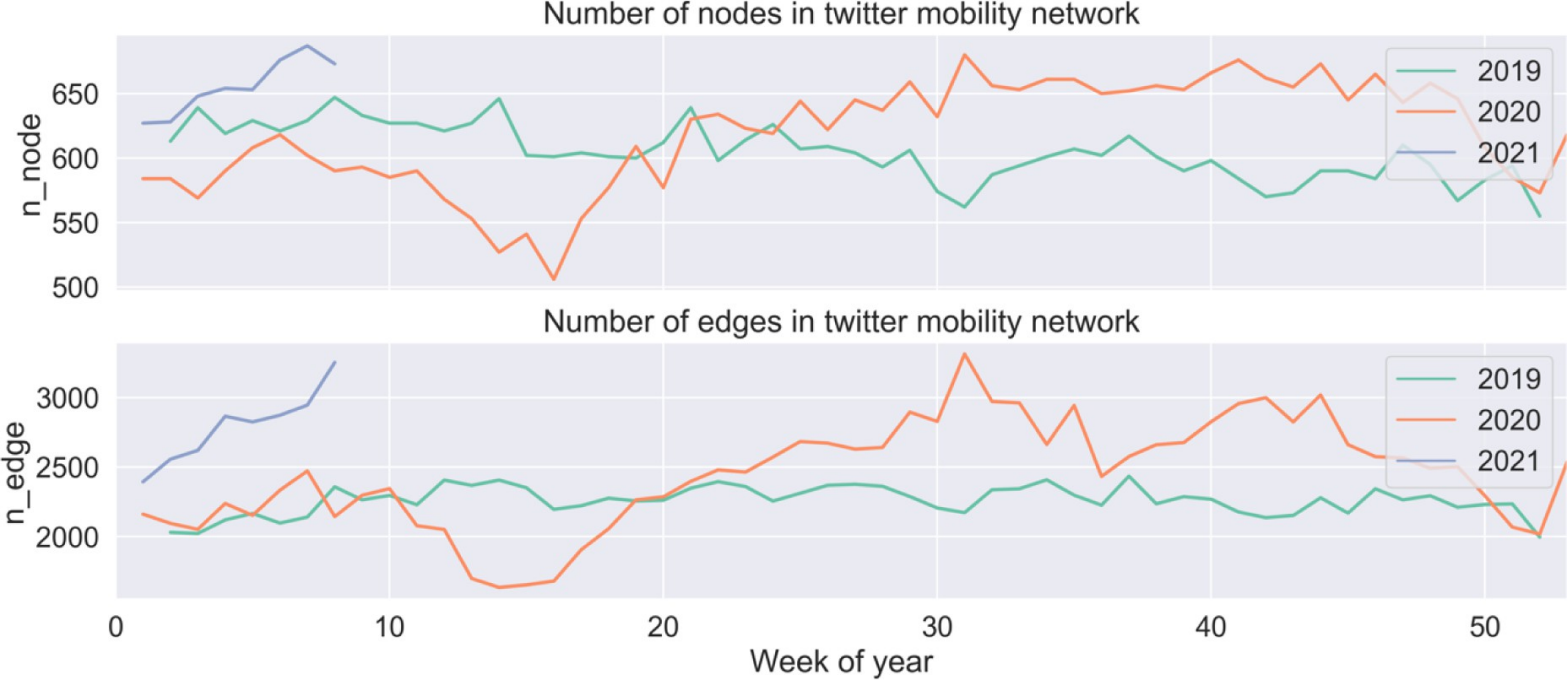
The number of connected nodes (MSOA zones) and edges (travels between zones).

Analysis of the spatial network by week allows us to generate a temporal profile of connectivity and attractiveness for each zone. To find out where people travel to, we have profiled a time series of connected nodes (MSOAs) and the edges, shown in Fig 6. The increased number of connected nodes and edges after March 2020 indicates an increasing variety of location choices. Our simple assumption is that these less favoured zones pre-covid become better location choices. Moreover, our statistics show a decreased rate of intra-zone trips (full statistics are given in S1 Table), despite an increased number of nodes and edges. It echoes our observation in section 4.1. that there is a comparatively longer travel distance than that pre-covid.

**Fig 7:**
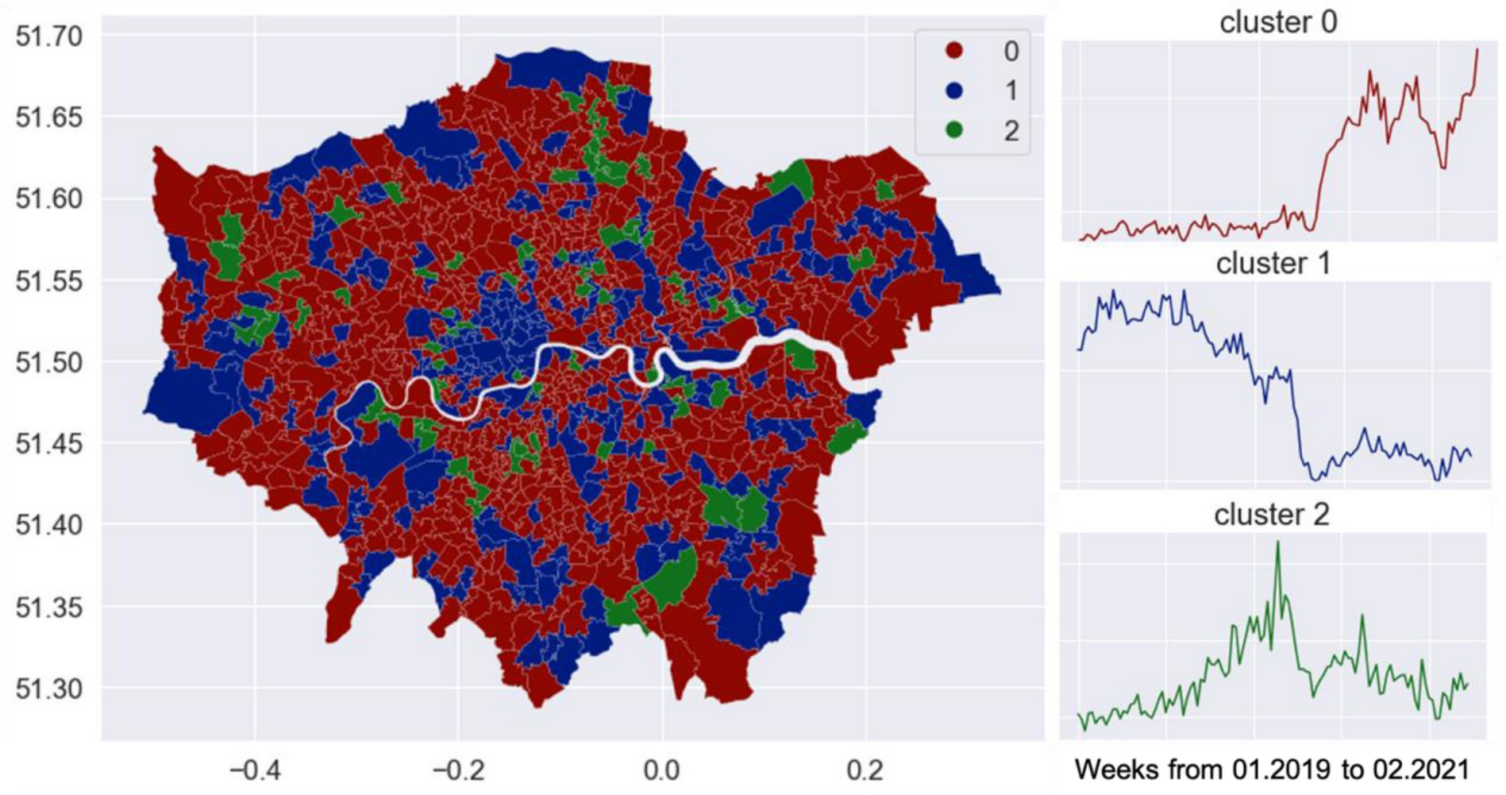
Changes in location attractiveness.

Instead of looking into each area, we grouped areas with a similar temporal profile. The spatial distribution of groups gives us an overview of the changes over space. There are in total 112 weeks from the week of 2019-01-07 to the week of 2021-02-22. That is, temporal profiles of the attractiveness were formulated as vectors of length 112. Agglomerative clustering is applied to group areas with a similar trend. In particular, hierarchical clustering uses a bottom-up approach. That is, each zone starts in its own cluster, and clusters are successively merged together. The similarity of zones is measured by Pearson correlations, rather than Euclidean distance. The optimal number of clusters is selected in support of a dendrogram visualisation. In the end, we decided upon three groups that best illustrate the variation in the temporal profiles as shown in Fig 7.

Again, reinforcing our finding of longer travel distance, some central areas (e.g. Camden town, and marked in blue as cluster 1) that used to be popular were less visited than in more normal days, and this is reflected by decreased values of degree centrality since March 2020 (shown in Fig 7). Instead, some of the peripheral areas (marked in red as cluster 0) got more visits, which is a rational choice since less dense areas were naturally associated with lower risk. Over the summer, when Covid measures were lifted, zones in all three clusters gained comparatively more visits. Nevertheless, overall number of visits is much less than that on the more normal days. The choice of location pre and during COVID-19 can be partially explained by the GLA spatial structure, that is, a dominant big urban centre–the West End is located in the geographical centre of the GLA and all the other scattered subcentres are comparatively very small and less attractive for Twitter reported activities.

**Fig 8:**
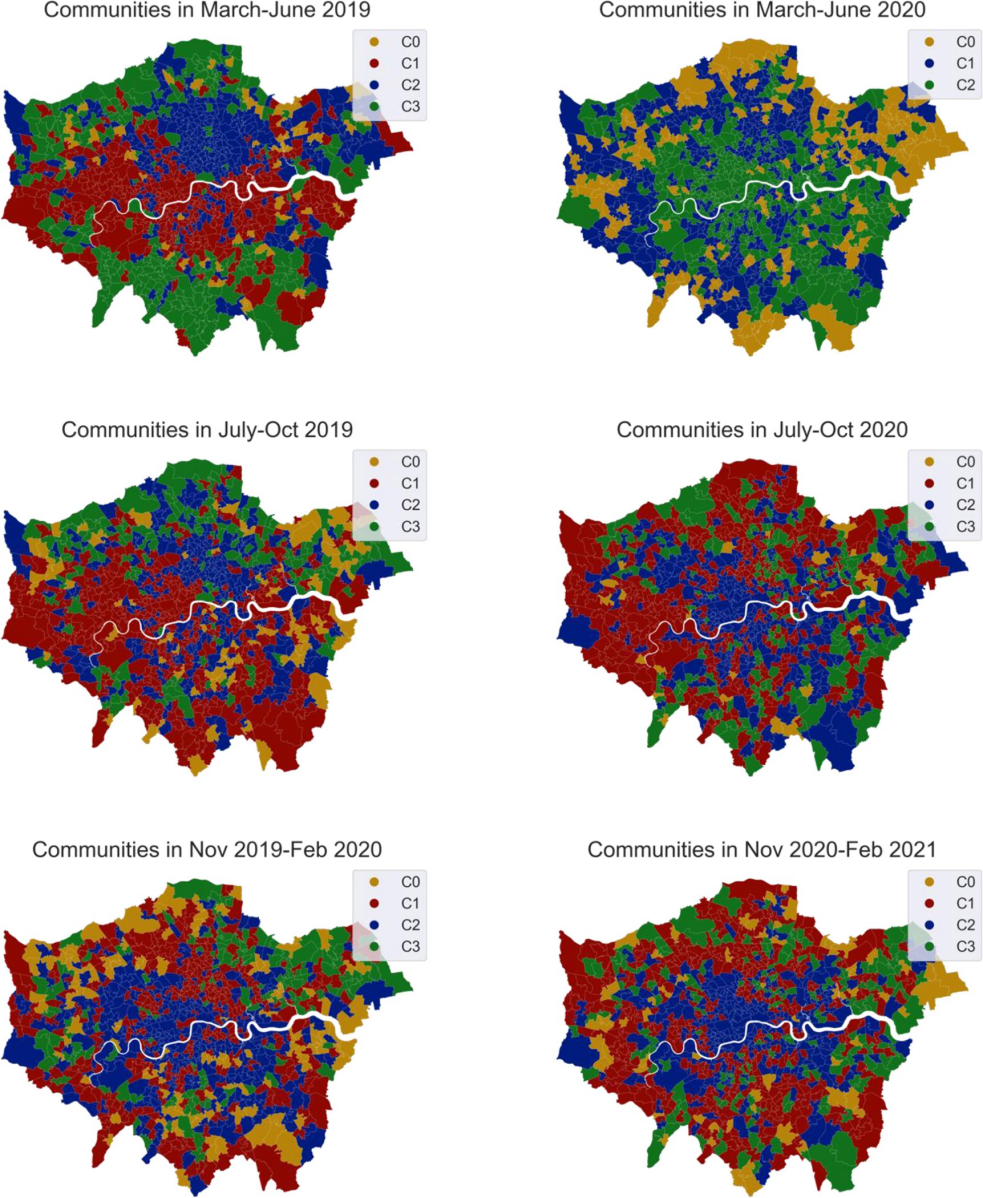
Changes in community structure.

Community detection was applied to spatial networks generated from three periods, March 2020 –June 2020 (first wave), July 2020 –October 2020 (summer period), November 2020 –Feb 2021(second wave), to look at the dynamic structure of inter and intra area trips over time. Modularity-based community detection is applied. Networks with high modularity have dense connections between the nodes within a community but sparse connections between nodes in different communities. In simple words, the MSOA zones grouped in one community are more closely connected to each other than that to the outside communities. There are always about three dominant communities detected in all periods. As shown in Fig 8, communities indexed C1, C2 and C3 are the biggest communities, composed of a large number of MSOA zones. The location of the community is highly in line with transport infrastructures pre-Covid, i.e., the community in March-June 2019 marked in green was distributed along the ring roads, that in red reflects more a distribution along public transport lines.

When comparing the community structure between pre-Covid (left column) and COVID-19 days (right column) in Fig 8, two clear changes are easily observed. One is the significant differences in overall structure, particularly from March 2020 to Oct 2020. It is, however, interesting to see that the pair of maps at the bottom show certain degrees of similarity, indicating, Twitter mobility, at least in relative terms, is gradually returning to normal patterns in terms of urban connectivity. Another change concerns the scattered yellow areas (C0), which are collections of several extremely small communities (i.e., communities with less than the standard deviation of the mean the number of members) They are viewed as isolated areas that are separated from the other parts of the city. During the first lockdown, some areas were even less visited or served as a self-contained town with less connection to the outside world. There are many more isolated areas present during the time of COVID-19 as expected but which have been re-connected to the dominant communities in recent months.

## 5 Discussion

### 5.1. Insights from the London case study

In this paper, we experimented with the use of geo-tweets for monitoring changes in mobility patterns with some interesting insights generated for the London case study. We demonstrated three simple mobility indices, namely the ratio of travelled users to the total geotagged tweeters and the average distance of travel, and a third–the local attractiveness at the MSOA level. All of these can be easily computed and used for continuously monitoring daily changes during and beyond COVID-19 at a relatively fine spatial and temporal scale.

We add a follow-up study here based on the above-defined indices to investigate (1) whether a strong relationship exhibits between the increased or decreased Twitter visiting zones and the number of Covid cases; (2) whether the transmission of Covid is caused by inter-zone travels or intra-zone activities or other local characteristics. To relate the Covid cases to changes in local attractiveness, we calculated Attractiveness difference by

$$attractiveness_{diff}=\frac{Degree(i,t)}{\sum Degree(i,t)}-\frac{Degree(i,t-1)}{\sum Degree(i,t-1)}$$ (4)

Where *i* is a node in the spatial network, which denotes an MSOA zone; t denotes the time period, i.e *t* is the year 2020 and *t*−1 is the year 2019.

**Fig 9:**
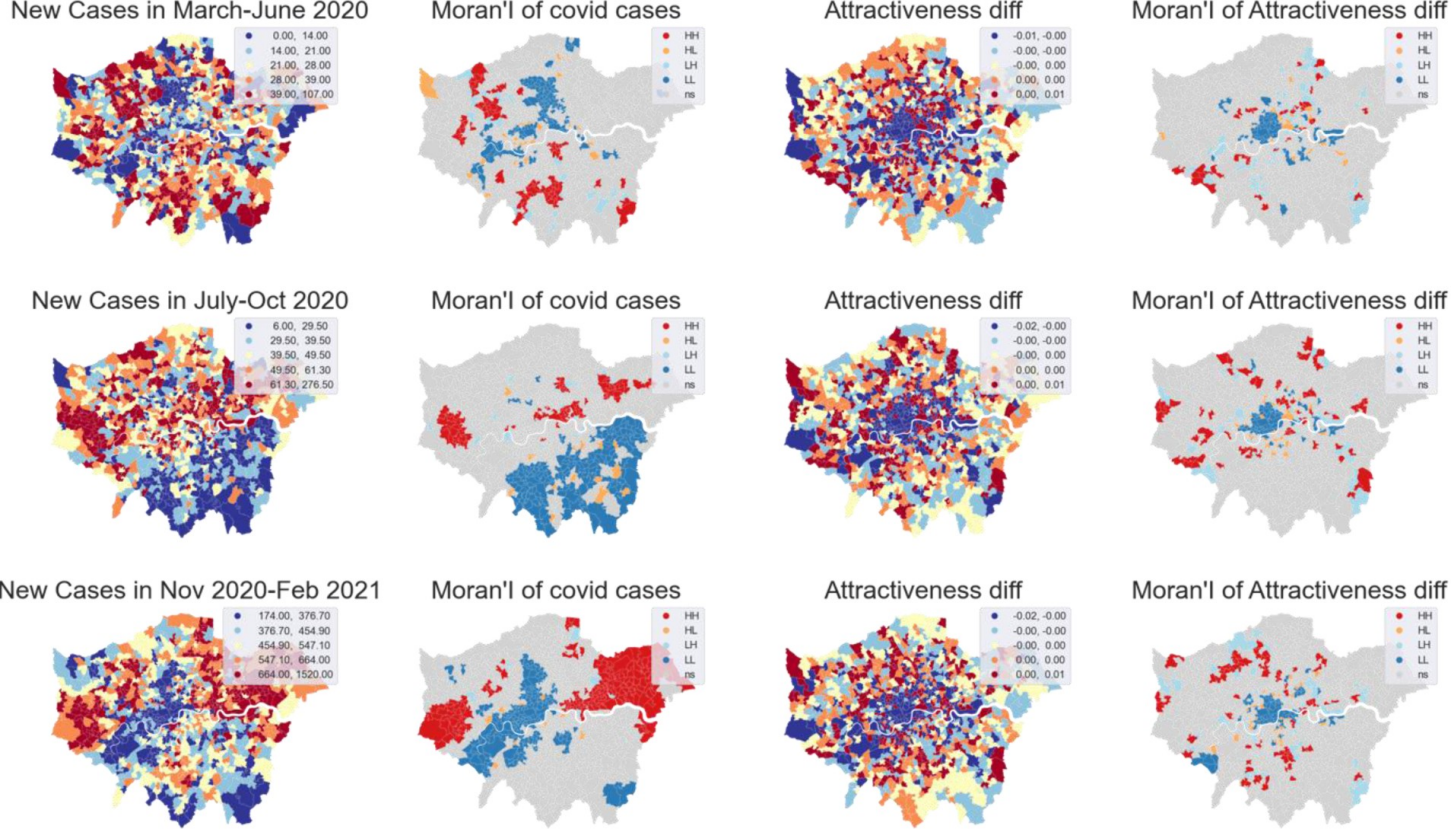
A visual comparison of spatial distributions. (Notes: rows from top to bottom are date in the three periods. The first column is the accumulated number of Covid cases during a given period; the second column shows a local Moran measure using a first-order queen weight matrix to highlight the concentration of Covid cases. The third and fourth columns show the differences in local attractiveness during the study period and the corresponding Moran I values.

**Fig 9** provides a visual comparison. Over the three periods, the reported cases of COVID-19 show a clear spatial autocorrelation. LL: Cold spots (blue areas where the reported number of Covid cases is low in clustered areas) and HH: red spots (red areas where the number of Covid case is high in clustered areas) are clearly shown. The spatial clusters generated from attractiveness difference are in line with our conclusion in the previous section that outer areas got increased visits during Covid. However, the spatial distribution shows little similarity to that of Covid cases, as shown in the local Moran’s I visualisation (**Fig 9** column 2 and 4).

**Fig 10:**
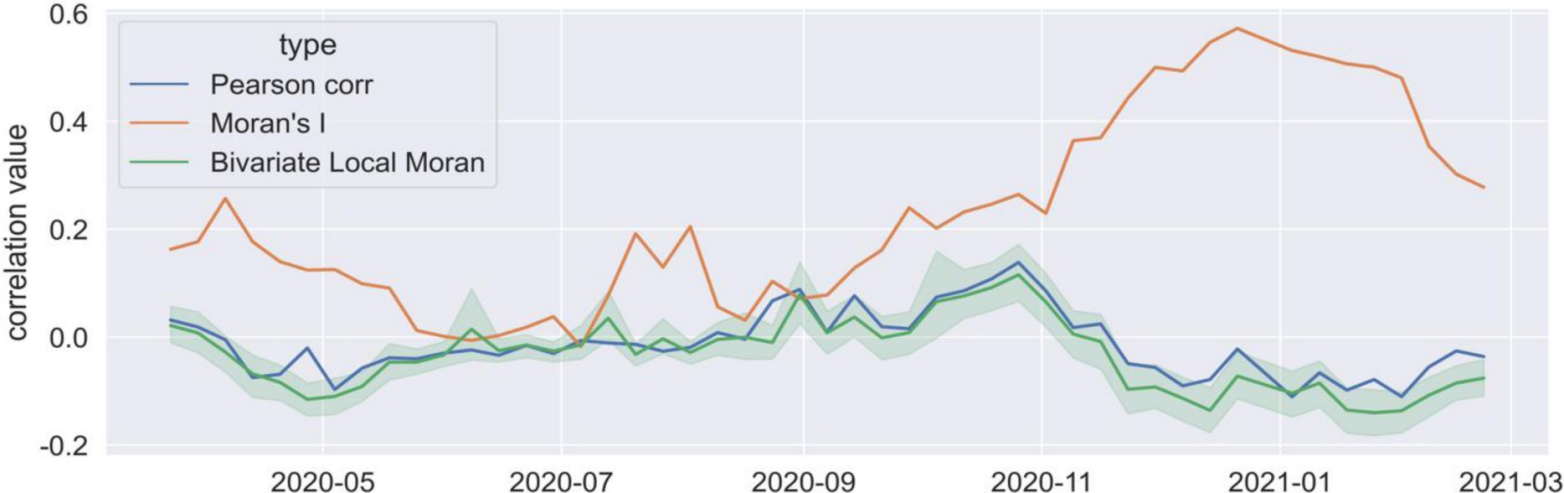
Summary of correlation measures.

In addition, a statistical comparison is presented in **Fig 10**, which summerised a series of correlation measures on a weekly basis. Reported Covid cases and local attractiveness were formatted as vectors with 983 dimensions (note that London has 983 MSOA zones). Four types of correlation measures were applied, which are (1) Pearson’s correlation measures their aspatial relationship; (2) Moran’s I measures the spatial autocorrelation of Covid cases; (3) the global bivariate Moran’s I measures the spatial relationship between Twitter mobility and Covid cases at time t, plotted as the dark green line; (4) the bivariate local Moran’s I identifies if any particular areas show exceptionally high or low correlations, plotted in the figure as a threshold in light green. The multiple peaks of Moran’s I are a global measure of the spatial clusters shown in **Fig 10**. Both aspatial and spatial correlations between attractiveness and Covid cases were constantly weak around 0, with negative correlations sometimes, and within the range of [-0.14, 0.12] over the entire study period. This indicates that little relationship was identified between Twitter mobility and the transmission of Covid cases among MSOA zones. More specifically, that means daily activities associated with journeys detected from geotweets cannot be the most crucial factor for Covid transmission in the case of London.

Overall, our case study shows that in tweet-rich areas, geo-tweets can be used as a valuable resource to monitor changes at a small spatial unit level. The analytical workflows presented in this work are relatively straightforward and implemented in Python by well-established packages. It is designed in such a way as to encourage the use of similar case studies in other cities for a comparative study. Given that cities have unique urban contexts, the findings from our case study may not apply either to the other cities or at different scales. Furthermore, compared to the automatic human mobility data, Twitter data is more accessible to the public and can be continuously collected relatively easily via public API with consistent data quality and standards. It could be used as an easily accessible monitoring tool during and beyond the current COVID-19 period.

More work could be developed beyond exploration and visualisation to deliver more comprehensive insights into disease transmission and its impacts on urban mobility and activity over population groups. First, as concluded in related literature on COVID-19 and different ethnic groups, in London and the UK, as well as in other countries, e.g. the US, minority ethnic groups and lower-income populations are like to get infected due to relatively higher exposure to COVID-19, through living in socioeconomically deprived, and overcrowded conditions [52, 53]. The opportunity to integrate our work with other mobility datasets and socioeconomic data suggests our future work should investigate more attributes in the correlation and spatiotemporal regression analysis to get a fuller picture of impact factors, generating a more detailed profile for local areas. Second, all these established mobility indexes use a pre-pandemic baseline and our proposed ones. An unspoken assumption is that some associated human behaviours (i.e., querying behaviour using Google maps, tweeting behaviour using Twitter) remain constant, which needs further investigation. Third, the causality between COVID-19 cases and mobility changes needs further investigation by a more comprehensive spatial-temporal modelling and considering all kinds of interventions.

### 5.2. Representativeness of Twitter mobility

**Fig 11:**
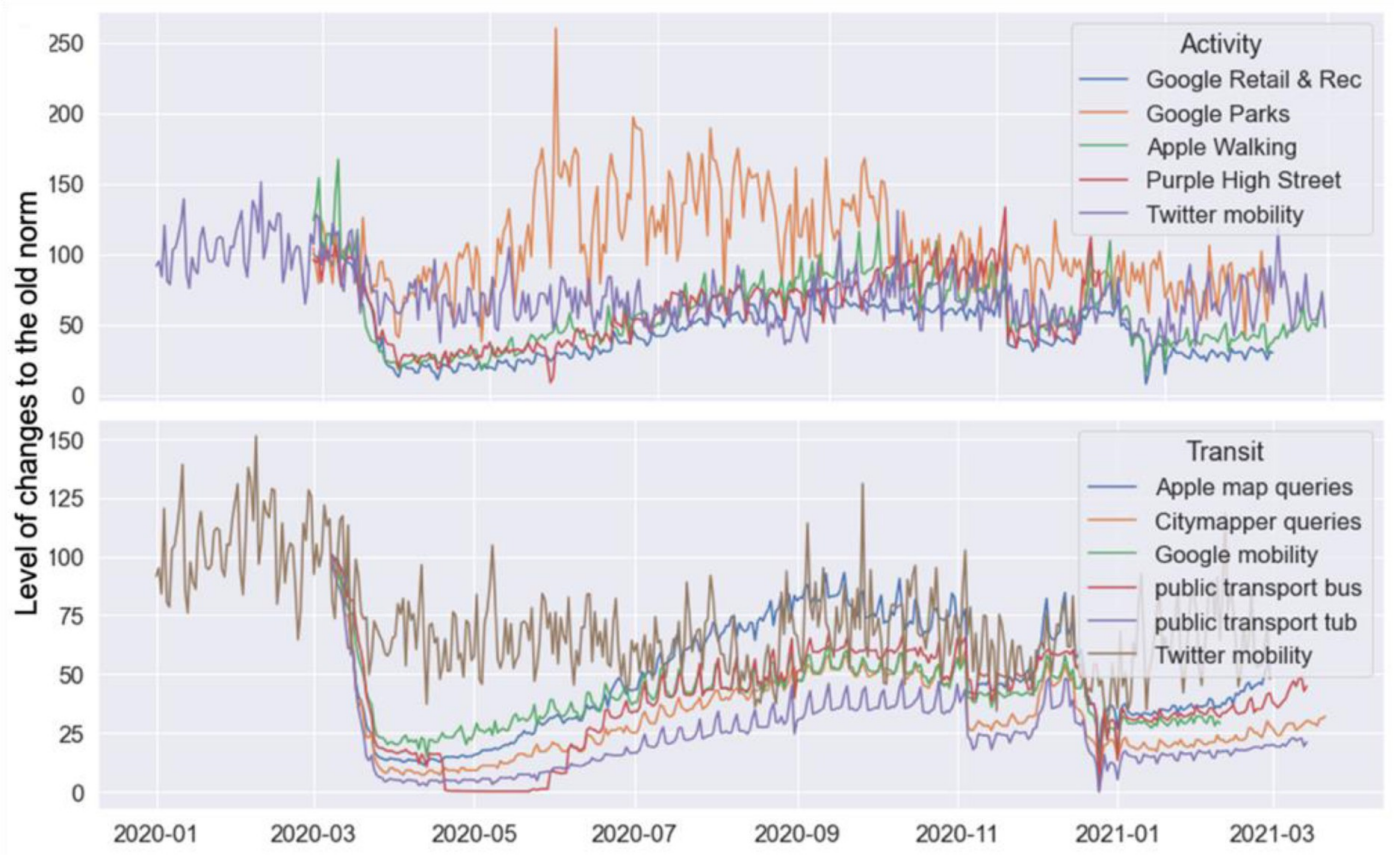
Comparison of Twitter mobility index (m_rate) to established mobility index (date source: https://data.london.gov.uk/dataset/coronavirus-Covid-19-mobility-report).

There is an ongoing discussion on the bias of Twitter data and its representativeness in the context of various applications. Previous work has discussed the bias, limitations and uncertainties of social media data from various angles, for instance, by comparing data from different platforms [54] and by critical analysis of methodology [55]. Since we are looking at relative changes rather than absolute values, the bias generated by the platform or methods should not exert significant impacts on the results. What matters most is the Twitter population and their motives for tweeting. The relevant research has suggested that Twitter users who publish geographical information are not representative of the wider Twitter population [56, 57]. Our previous analysis also suggests that centres revealed by geo-tweets are economic clusters rather than multi-functional ones [26] and the topics extracted from tweets are more about social life. To further investigate the representativeness of the detected Twitter mobility, we compared m_rate(coviddays)/m_rate(pre_covid) to the other established ones summarised in the GLA (Great London Authority)’s Coronavirus (COVID-19) Mobility Report (https://data.london.gov.uk/dataset/coronavirus-covid-19-mobility-report). Briefly, their established Twitter mobility index is calculated based on activities and transit extracted from geotagged queries made by Google, Apple, and Citymapper apps and is linked to urban features. The extracted intensity of activity and journeys were reported (in Fig 11) as a percentage of a pre-pandemic baseline. By plotting these indices together, we found that the overall trend shares certain degrees of similarity. In particular, the overall trend of mobility went down since the first lockdown and increased a little overall during the summer. However, as shown in Fig 11, the Twitter mobility index does not show any high levels of overlap with any of these established measures. In particular, Twitter mobility does not reflect the same level of transit demand drop when Covid measures were first established in March 2020. Comparatively, Twitter mobility is a better proxy of urban activities rather than transit usage and as an averaged intensity of multiple social activities (e.g., Highstreet and walking for exercise), with a moderate correlation between Twitter mobility and high street visits (0.46) and walking (0.50). A full correlation matrix can be found in S3 Fig. The underlying reason is rooted in the way the data was generated. Who, for instance, is using the service [54]? And for what purposes? We, therefore, concluded that the proposed Twitter mobility indices are a measure of social activity, rather than transit demand, that is better used for monitoring economic recovery and changes in lifestyles.

Twitter data bias is a long-standing issue that needs to be further addressed in different application contexts. In particular, the spatial distribution of tweets strongly varies on different spatial scale levels and might be too sparse in some geographical areas [58]. Our work attempted an analysis at the MSOA level and was limited to inter-MSOA travel within the GLA. Because Covid measures might have exerted a much more significant impact on inter-city travels (e.g., London to Cambridge) and inter-country travels. That means the conclusion drawn on the relationship between COVID-19 cases and Twitter mobility may alter at a different spatial scale. This will be investigated in our future work on data uncertainty across spatial and temporal scales with a similar mobility indicator-based research methodology. Furthermore, regarding the data uncertain issue, Huang, Li (34) has compared various mobility data sources, revealing unique and contrasting characteristics in mobility patterns in response to COVID-19 in the U.S at the county level. This work shows that the Twitter mobility index is sensitive to Covid-induced mobility. Therefore, Twitter could be used as an effective tool for monitoring mobility changes. In [32], movements detected from Twitter data have been compared with those derived from mobile phone and Facebook data in the US case study. They found positive correlations between these different measures using different data sources. However, one cannot substitute one for the other, as they represent various phenomena due to the nature of the data. Another study in the UK used Facebook data to monitor the impact of lockdown on travel distance and flow volumes at a regional scale [18]. Continuously changed mobility patterns were observed like that in our study, but they also mention possible date uncertainty in Facebook data capturing and processing. Furthermore, the Twitter data policy change has impacted the Twitter data quality and for research use [59]. Whether previous work could be reproduced with future Twitter data sets in reduced volume and location accuracy is questionable. Nevertheless, all these suggested the need to investigate further the representativeness of mobility data across spatial and temporal scales by contrasting and combining multisource data with a uniform analytical framework.

## 6 Conclusion

COVID-19 has caused dramatic effects on everyone’s life. It limited our physical activities and social interaction, as shown by our analysis of daily travels and community interaction. On the other hand, it has accelerated the digitalisation process and the emergence of new digital products such as data. During COVID-19, various nationally publicly available data sets have been generated for current and future disease outbreaks and prevention. Other than most of the data analysis and visualisations at country or city scales, we challenged the use of freely available human location data (i.e., geotweets in this work) for tracking smaller-scale changes. Tweets are indeed not an ideal data set, as discussed in our work and many relevant papers, but with effective indicators and analysis, some collective effects can still be detected. In fact, there are no perfect big data sets. In the discussion, we have compared changes detected in tweets to that extracted from other data sources. The results do not show a high consistency among any two data sets. That indicates the direction of our future work, which is about the reliability and uncertainty of emerging human location data sets. Beyond the use case of COVID-19, more research shall be taken to develop a uniform analytical framework.

## Supporting information

S1 Fig Number of geotweets and extracted twitter trips in year 2019, 2020, and 2021. The overall spatial distribution shows certain degrees of similarity. The intensity of extracted travels and counts of collected geotweets shows a good level of correlation, which is further detailed in S2 Fig.
(TIF)

S2 Fig Correlation matrix of tweets count, detected visits, population and population density at MSOA level. Count2019, count2020, count2021 are the number of geotweets collected. Degree2019, degree2020, and degree2021 are the number of travels to an area. As it shows (1) comparatively very low correlation between Twitter measures (counts and degrees) between population and density, which indicates the tweeting behaviour has little relationship with commuting trips from residential areas. (2) Degree2019 has a low correlation with degree2020 and degree2021, which indicates changes in location choices during COVID-19.
(TIF)

S3 Fig Correlation matrix of weekly twitter mobility and Google reported activities. (TIF)

S1 Table Number of nodes and edges in the weekly network(top) and seasonal network(bottom). On average, the number of connected zones increased, in contrast, and the intra-zone travels decreased.
(TIF)
