# Supplementary figures and images for: Twitter mobility dynamics during the COVID-19 pandemic: A case study of London
Source: PLoS One. 2023 Apr 26;18(4):e0284902. doi: 10.1371/journal.pone.0284902 (PMC10132666; doi:10.1371/journal.pone.0284902)

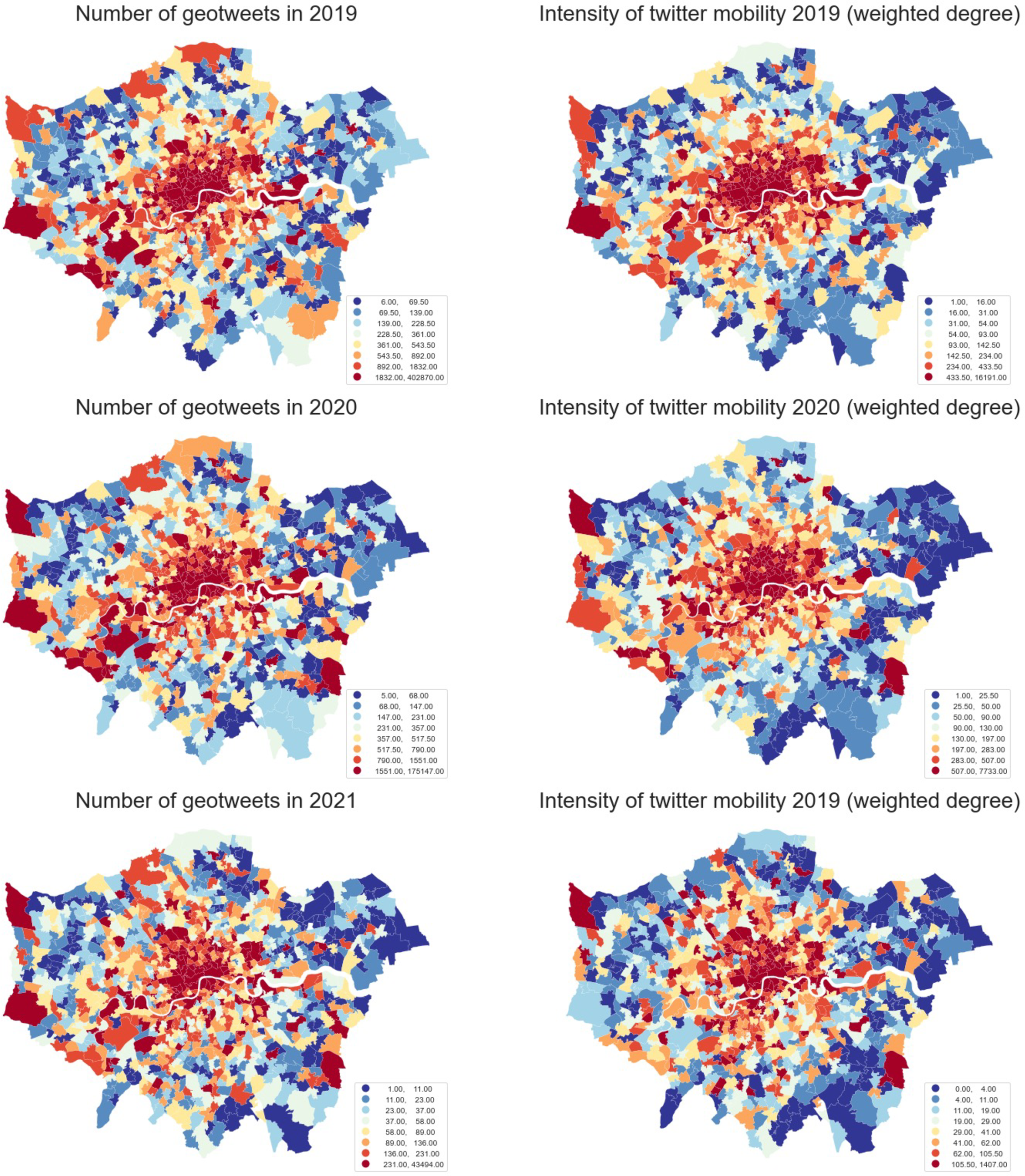

Supplement: S1 Fig — The overall spatial distribution shows certain degrees of similarity. The intensity of extracted travels and counts of collected geotweets shows a good level of correlation, which is further detailed in S2 Fig. (TIF) [file pone.0284902.s001.tif]

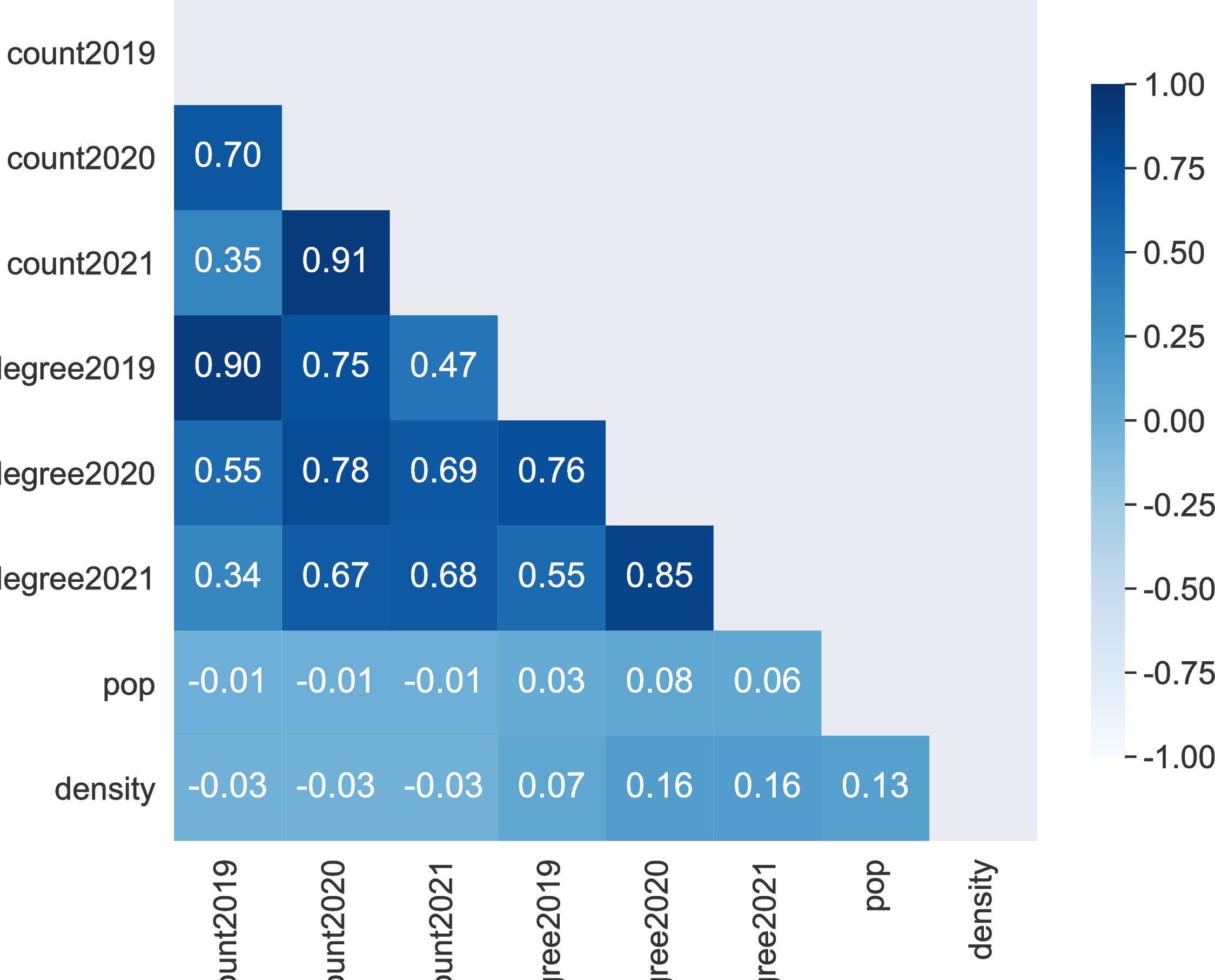

Supplement: S2 Fig — Count2019, count2020, count2021 are the number of geotweets collected. Degree2019, degree2020, and degree2021 are the number of travels to an area. As it shows (1) comparatively very low correlation between Twitter measures (counts and degrees) between population and density, which indicates the tweeting behaviour has little relationship with commuting trips from residential areas. (2) Degree2019 has a low correlation with degree2020 and degree2021, which indicates changes in location choices during COVID-19. (TIF) [file pone.0284902.s002.tif]

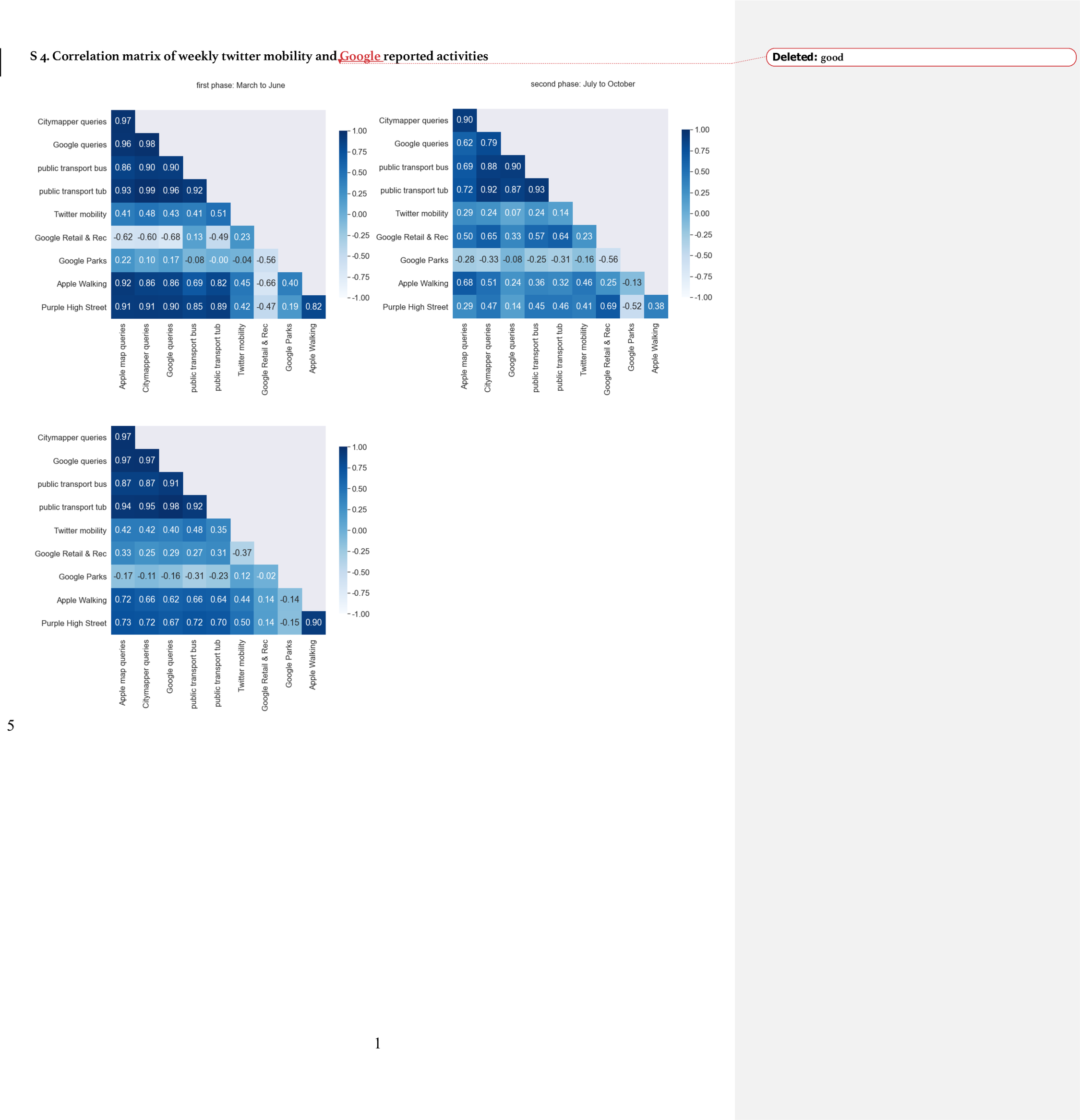

Supplement: S3 Fig — (TIF) [file pone.0284902.s003.tif]

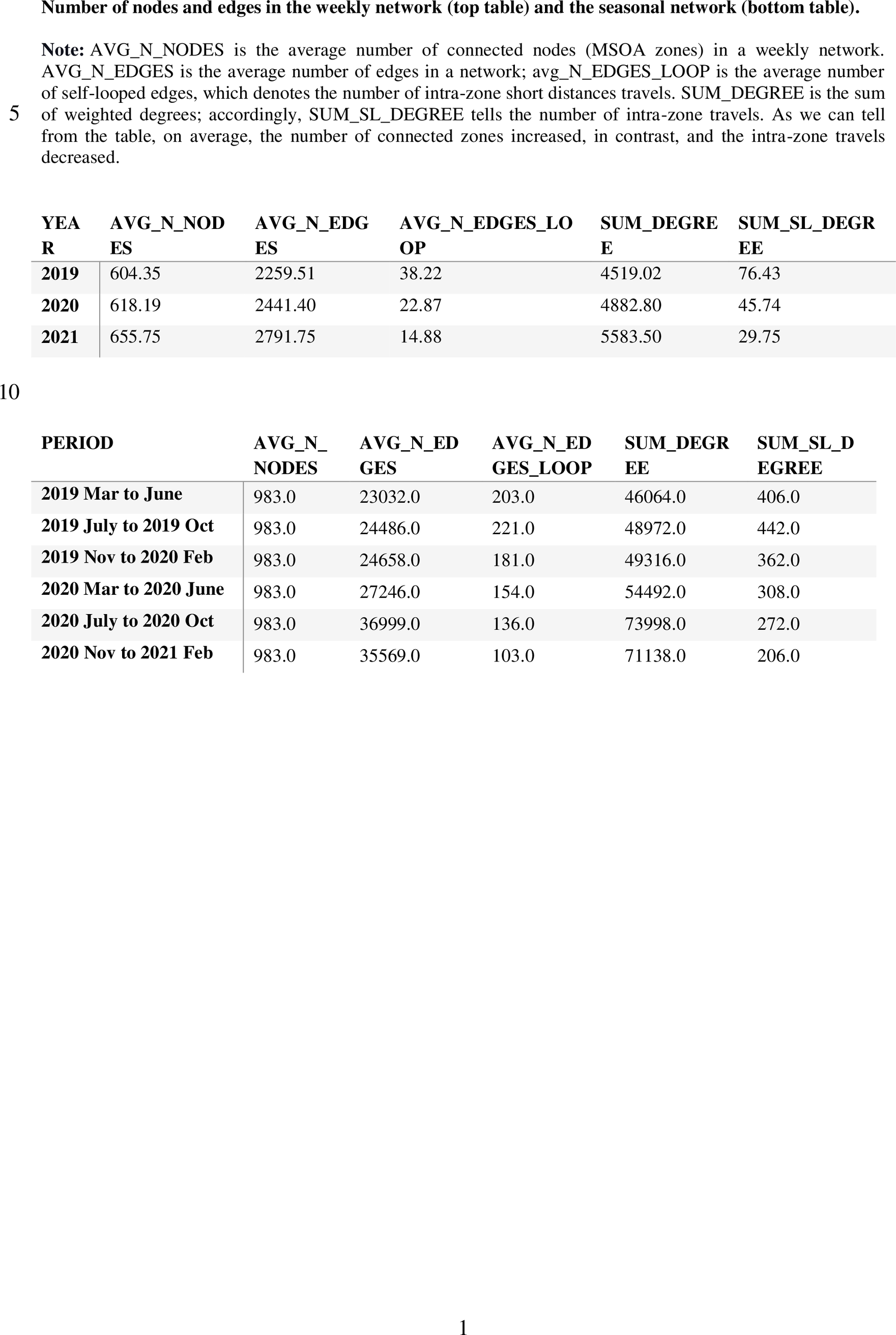

Supplement: S1 Table — Number of nodes and edges in the weekly network(top) and seasonal network(bottom). On average, the number of connected zones increased, in contrast, and the intra-zone travels decreased. (TIF) [file pone.0284902.s004.tif]
